# Neutrophils, Cancer and Thrombosis: The New Bermuda Triangle in Cancer Research

**DOI:** 10.3390/ijms23031257

**Published:** 2022-01-23

**Authors:** Mélanie Langiu, Ana-Luisa Palacios-Acedo, Lydie Crescence, Diane Mege, Christophe Dubois, Laurence Panicot-Dubois

**Affiliations:** 1 Aix Marseille Univ INSERM, INRAE, C2VN, 13005 Marseille, France; melanie.langiu@univ-amu.fr (M.L.); ana-luisa.palacios-acedo@univ-amu.fr (A.-L.P.-A.); Lydie.crescence@univ-amu.fr (L.C.); diane.mege@univ-amu.fr (D.M.); laurence.panicot-dubois@univ-amu.fr (L.P.-D.); 2 Department of Digestive Surgery, La Timone University Hospital, 13005 Marseille, France

**Keywords:** cancer, thrombosis, neutrophil, NET

## Abstract

Spontaneous venous thrombosis is often the first clinical sign of cancer, and it is linked to a worsened survival rate. Traditionally, tumor-cell induced platelet activation has been the main actor studied in cancer-associated-thrombosis. However, platelet involvement alone does not seem to be sufficient to explain this heightened pro-thrombotic state. Neutrophils are emerging as key players in both thrombus generation and cancer progression. Neutrophils can impact thrombosis through the release of pro-inflammatory cytokines and expression of molecules like P-selectin and Tissue Factor (TF) on their membrane and on neutrophil-derived microvesicles. Their role in cancer progression is evidenced by the fact that patients with high blood-neutrophil counts have a worsened prognosis. Tumors can attract neutrophils to the cancer site via pro-inflammatory cytokine secretions and induce a switch to pro-tumoral (or N2) neutrophils, which support metastatic spread and have an immunosuppressive role. They can also expel their nuclear contents to entrap pathogens forming Neutrophil Extracellular Traps (NETs) and can also capture coagulation factors, enhancing the thrombus formation. These NETs are also known to have pro-tumoral effects by supporting the metastatic process. Here, we strived to do a comprehensive literature review of the role of neutrophils as drivers of both cancer-associated thrombosis (CAT) and cancer progression.

## 1. Introduction

The connection between cancer and thrombosis has been described since the XIX century by Dr Trousseau who described the presence of spontaneous coagulation in oncological patients [1,2]. Cancer patients have an increased incidence of both venous thromboembolism (VTE) (4% to 20%) and arterial thrombosis (2% to 5%) [3,4]. In fact, between 20 to 30% of all first venous thrombotic events are cancer related, and their presence portends poor prognosis and a significant decrease in patient survival [5,6]. The incidence of cancer-associated-thrombosis (CAT) is also associated with the tumor type, stage and treatment administered. CAT is the second most common cause of death in cancer patients (after cancer evolution itself) and thrombi can be found in half of deceased cancer patients during autopsy [1,7,8,9].

Platelets are at the crossroads of hemostasis, thrombosis, and inflammation. As such, their involvement in CAT has been largely investigated. Cancer cells can activate platelets through tumor cell induced platelet activation (TCIPA) [2]. In a direct manner, platelet receptors like αIIbβ3 and αVIβ1 can bind to tumor αVβ3 and ADAM9 respectively, as well as binding with P-selectins through PSGL-1 interaction, platelet toll-like receptor 4 and facilitating CLEC2-podoplanin interactions [10,11,12,13,14]. In an indirect manner, tumor cells secrete platelet agonists (like thromboxane A2 and ADP) into the tumor microenvironment, activating platelets which then release more platelet agonists and create a potent activation loop [11,12,13,15]. Tumor-activated platelets can also secrete growth factors into the tumor microenvironment like transforming growth factor beta (TGF-β), vascular endothelial growth factor (VEGF), and platelet derived growth factor (PDGF) [2,13,16]. This platelet degranulation can also support local angiogenesis and increase the endothelial expression of adhesion molecules [13,17,18]. This overall increased platelet activity is related to a heightened risk of venous and arterial thrombosis in cancer patients [2,19,20,21].

However, platelet involvement alone does not seem to be sufficient to explain the heightened pro-thrombotic state in cancer patients. Recently, neutrophils have been beginning to enter the spotlight for their role in both tumor progression and CAT. Indeed, recent studies described that an increase in circulating neutrophil numbers was indicative of a worsened prognosis in gliomas, lung, and esophageal cancer [22,23,24]. To date, the relevant molecular mechanisms are not entirely clear into the literature. In this review will strive to compile the current state of the art of neutrophil involvement in cancer progression and in cancer-associated thrombosis.

## 2. Neutrophils and Thrombosis

Neutrophils are the most abundant leucocytes in humans and constitute an important pillar of the innate immunity [25,26]. They are the first immune cells to be recruited to inflammatory sites [25,26]. Neutrophils are also key players in intravascular immunity; their microbicidal activity prevents the spread of circulating pathogens [25]. Neutrophils have the capacity to mediate phagocytosis and intracellular killing of different pathogens as well as eliminate cellular debris in their phagolysosomal granules [25,27,28].

Circulating neutrophils are recruited to extra-vascular inflammatory sites via a chemokine gradient [29]. Studies have shown that neutrophils react preferentially to a hierarchy of chemokines, allowing them to reach the desired endothelial placement and then transmigrate to the surrounding tissue [29]. For example, bacterial-derived N-formyl-Methionyl-leucyl-phenylalanine (fMLP) and complement C5a override CXCL8/IL-8 and LTB4 chemotactic stimuli [29,30]. The actual recruiting cascade involves several well characterized steps: rolling-adhesion-tethering-crawling and transmigration. The endothelium is stimulated by inflammatory mediators like histamine and cytokines or pattern-recognition-receptors that will enhance P-selectin and E-selectin expression on the endothelial-cell surface to maximize neutrophil recruitment [29]. Neutrophils will begin “rolling” on the endothelial surface before being primed and activated by molecules like tumor-necrosis-factor-α (TNFα) as well as by direct contact with activated endothelial cells [29]. Priming of the neutrophils is necessary to achieve activation of the NADPH oxidase pathway used to destroy pathogens [29,30,31]. As the neutrophils slow down, they “crawl” between endothelial cells to reach the cell-cell junctions via ICAM1-MAC1 signaling [29,30]. Eventually neutrophils tether to the endothelium before transmigrating, a complex process that requires multiple integrin interactions (ICAM1 and 2, VCAM1, PECAM1 and EPCAM to name a few) [29]. Transmigration can occur between endothelial cells or transcellularly, and once neutrophils are in the tissue, they can zoom-in on the inflammatory site [26,29,30].

Neutrophils can eliminate pathogens in different ways; in both an intra and extracellular manner [27,31]. They can internalize opsonized pathogens or cellular debris into their phagolysosomal granules for destruction with reactive oxygen species (NADPH oxidase pathway) [29]. They also secrete their granule content to combat extracellular pathogens [29,31,32]. Neutrophils have three distinct granule types: **(1) Azurophilic or primary** containing myeloperoxidase (MPO), cathepsin G, neutrophil elastase (NE) and bactericidal/permeability increasing protein (BPI) [31,32,33]. **(2) Specific or secondary granules** with alkaline phosphatase, NAPDH oxidase, collagenase, lactoferrin and histaminase and **(3) the tertiary granules** with collagenase, cathepsin and gelatinase [29,31,32,33].

The role of neutrophils in the innate immunity is well characterized; however, their importance in other phenomena like thrombosis and hemostasis has only recently begun to be determined. It is also clear that in an altered pathological state like cancer, most of the known mechanisms can be heightened, creating an amplification loop. The role of neutrophils in thrombosis began to be elucidated in 2005 when it was reported that leukocytes were rolling onto the growing thrombus site within the first two minutes post laser injury [34]. However, it was not until 2012 that the importance of neutrophils in thrombus formation was cemented [35]. Indeed, it was shown by Darbousset et al. that mice neutrophils were the first cells to accumulate at the site of arterial injury, even before platelets were detected [35]. Neutrophils accumulate through the interaction of intracellular-adhesion-molecule-1 (ICAM1) with leukocyte-function-associated-antigen-1 (LFA1) and express TF [35]. Moreover, neutrophil depletion has been shown to have a significant attenuation effect on thrombus formation in mice in vivo [36]. Interestingly, soluble fibrinogen found in prothrombotic conditions can also activate neutrophils in a CD11-b dependent mechanism which contributes to an increase in proinflammatory signals [37].

Neutrophils are a crucial leukocyte subset recruited at the site of injury, in fact, they release neutrophil elastase and augment their intracellular calcium mobilization, evidencing their activated state [38,39,40]. Additionally, ATP secreted during the thrombo-inflammatory process can directly activate neutrophils through the P2X1 membrane receptor [38]. It has been shown that P2X1 present on PMNs is involved in thrombin generation, and that its expression at the surface of platelets is needed for thrombus formation [38]. Several researchers, like Darbousset, R. et al. have shown the effect of ATP on thrombosis; and how it can activate human neutrophils in vitro [38]. On the other hand, in in vivo studies, P2X1-deficient mice had less neutrophil-accumulation and decreased thrombus formation at a laser-induced-injury site [38]. P2X1 activation leads to both platelet and neutrophil activation and activated platelets will then secrete their granules containing ADP, which enhances the platelet activation at the thrombus site [2,38,41]. These platelets will undergo intracellular calcium mobilization and adhere to the forming thrombus [42].

Another possible route in which neutrophils can impact thrombus formation is through the direct expression of TF on their membrane; this TF can be “obtained” after the neutrophil interacts with monocytes and/or macrophages [28,43]. Neutrophils that are first to arrive to the site of a laser injury can also express TF on their surface after interaction with endothelial cells [35]. More specifically, human neutrophils can express TF after stimulation with P-selectins and fMLP [44]. In inflammatory conditions, IgG triggers complement activation and C5a generation, which can induce TF gene transcription in activated human neutrophils [45].

**Figure 1:**
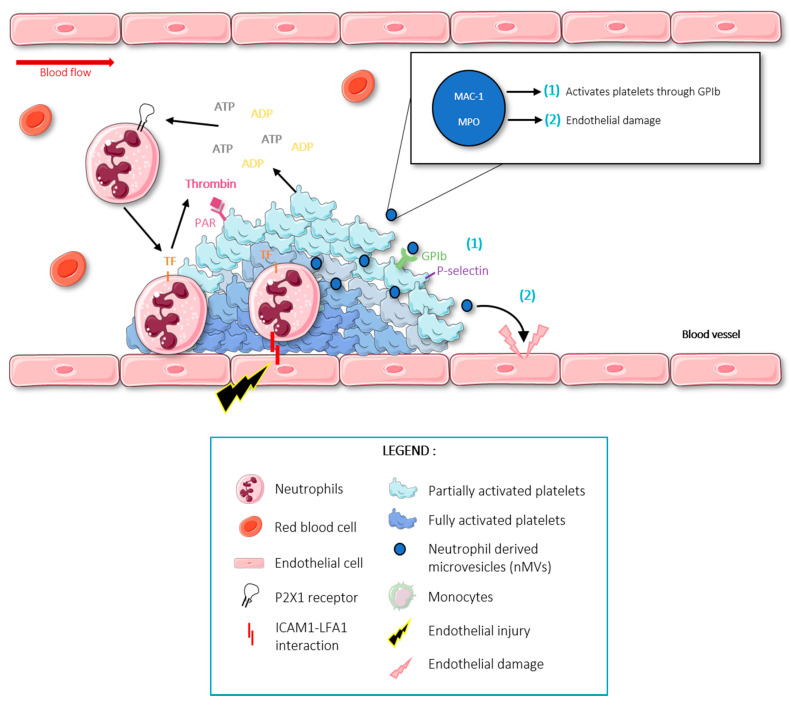
Schematic representation of neutrophil interactions and implications in thrombosis. Following endothelial injury, activated neutrophils express TF, which initiates the coagulation cascade resulting in thrombin generation and platelet activation. Activated platelets create a positive feedback loop to recruit more circulating platelets. Figure created using Servier Medical Art available at http://smart.servier.com/ (accessed on 15 September 2021).

A proposed molecular mechanism through which neutrophils impact thrombosis is through the generation of microvesicles (MVs). Microvesicles are small membrane vesicles released during cellular activation that contain the same membrane markers and proteins as the cell of origin [46,47,48]. MVs are known to have important contributions in inter-cellular communications and in pathological conditions like cancer [48,49,50]. MVs express phosphatidyl-serine on their outer membrane, which not only facilitates formation of coagulation complexes, but also promotes the ability of tissue factor (TF) to initiate coagulation [51]. Moreover, neutrophil-derived MVs may contain MPO, which has been shown to play a role in thrombus propagation by causing endothelial damage [28,29,30,31,32,33,34,35,36,37,38,39,40,41,42,43,44,45,46,47,48,52]. Human Neutrophil-MVs obtained after a PMA stimulation contain functionally active MAC-1 integrins that interact with GPIb on resting platelets. This interaction can activate platelets and induce the expression of the adhesion molecule P-selectin [53]. Please find a schematic revision of the role of neutrophils in thrombosis in Figure 1.

## 3. Neutrophil Extracellular Traps or Activated Neutrophils in Venous Thrombosis

**Figure 2:**
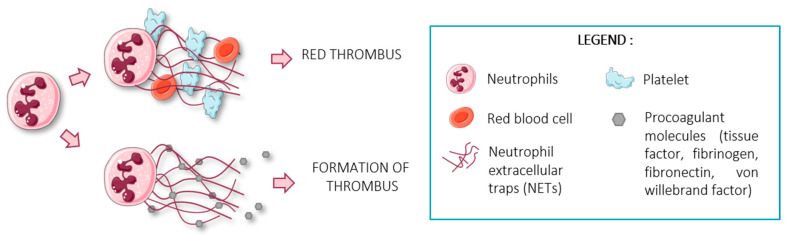
Schematic representation of NET interactions and implications in thrombosis. NETs are capable of trapping platelets and red blood cells, forming a red thrombus. In addition, they express pro-coagulant molecules such as tissue factor, fibrinogen, fibronectin or Von Willebrand factor, which initiate the coagulation cascade and participated to the thrombus formation. Figure created using Servier Medical Art available at http://smart.servier.com/ (accessed on 15 September 2021).

The role of neutrophil extracellular traps (NETs) in thrombosis has been recently described (Figure 2). NETs are part of the innate immunity’s defense against pathogens in which the neutrophil sacrifices itself by expulsing its genetic material, thus forming «neutrophil extracellular traps» (NETs) [54]. 

NETs are fibers of decondensed chromatin (DNA and histones) coated with antimicrobial proteins (myeloperoxidase (MPO), cathepsin G and neutrophil elastase (NE)) that are released by the neutrophil when it detects pathogens (bacteria, protozoa, fungi) in the extracellular environment [54]. These chromatin fibers create a network that entraps pathogens, preventing their dissemination in the host organism and eliminating them due to their anti-microbial properties [54,55]. 

The reaction of NET formation is catalyzed by peptidyl-arginine deiminase 4 (PAD4); which enables the chromatin de-condensation; causing Histone 3 to be citrullinated (CitH3) [56]. Therefore, by detecting either CitH3 or extracellular DNA, researchers can identify NETs. Indeed, the involvement of NETs in thrombosis was demonstrated when CitH3 was found in the thrombi of patients with VTE, and it was shown that the dissociation of NETs could promote the lysis of the thrombus [57].

Interestingly, studies have also highlighted the role of the enzyme PAD4 in the involvement of NETs in thrombosis [58]. Indeed, Martinod and colleagues demonstrated that after 48 h of inferior vena cava ligation only 10% of PAD4-deficient mice developed thrombi versus 90% in wild-type mice [58]. So PAD4 appears to be crucial in pathological venous thrombosis formation. Furthermore, no NETs were found in the thrombi from the PAD4-deficient mice, further highlighting their role in thrombus formation [55].

The network formed by NETs to trap pathogens can also trap platelets, an essential player in thrombosis [59]. In fact, Fuchs and colleagues have shown that NETs provide a scaffold for the activation and aggregation of platelets, as well as for red blood cells which form the red portion of the thrombus [59]. These data are supported by the fact that when mice were injected with DNase-I (a DNA-cleaving nuclease), NETs were degraded and no platelet aggregates were formed [59]. This suggests that NETs form an essential pro-thrombotic substrate for thrombosis.

The interaction of platelets and molecules that are entrapped in the NETs like von Willebrand factor (vWF), fibronectin and fibrinogen can also induce platelet aggregation [59]. The presence of fibrinogen causes the clots to consolidate through its transformation into fibrin in a thrombin-dependent manner [59]. In fact, Longstaff and colleagues have shown that contact between NETs (DNA + histones) and fibrin induces thicker fibers with improved stability and stiffness, and that the combination of histones and DNA significantly prolongs clot lysis time [60].

Additionally, Stakos and colleagues demonstrated that NETs were capable of secreting functional tissue factor (TF) [61]. TF is the initiating molecule of the extrinsic coagulation pathway and ultimately leads to thrombus formation [62]. The chromatin fibers can also inhibit the inhibitors of the extrinsic pathway, resulting in an over-activity of this coagulation pathway [63].

Nevertheless, Carminita et al. have demonstrated that following laser-induced injury, neutrophils -but not NETs- are involved in thrombus formation [41]. In fact, activated neutrophils already express CitH3 and PAD4, confirming that they are markers of neutrophil activation rather than NET formation [41]. Moreover, they showed that the inhibition of thrombus formation by DNase-I could be independent of NET formation [41]. This shows the current need for more research on the real role of NETs in thrombus formation.

## 4. Neutrophils in Cancer Development

Neutrophils can play a role in the development of cancer. This is highlighted in the relationship between neutrophilia and worsened prognosis in oncological patients [64]. Cancer cells can secrete chemokines, like IL-8, as well as GRO chemokines (CXCL1/2/3) and TGF-β to induce neutrophil migration to the primary tumor [64,65]. This chemokine explains why tumor-associated neutrophils (TANs) are localized at the margins of the tumor site in the early stages of cancer and may then massively infiltrate the tumor center in advanced stages [64].

Neutrophil secretion of chemokines is also enhanced during cancer development [66,67]. Cytokine IL17 can impact the tumoral microenvironment and cause the tumoral stroma to develop pro-tumorigenic functions, indeed Hayata et al. showed that in a mouse model inhibition of IL17a actually increased the cytotoxicity of tumor-infiltrating lymphocytes [66,68]. They also participate in immune cell recruitment to the tumor and enhancing the cancer-associated inflammation and promoting pathogenic T-cells [67].

**Figure 3:**
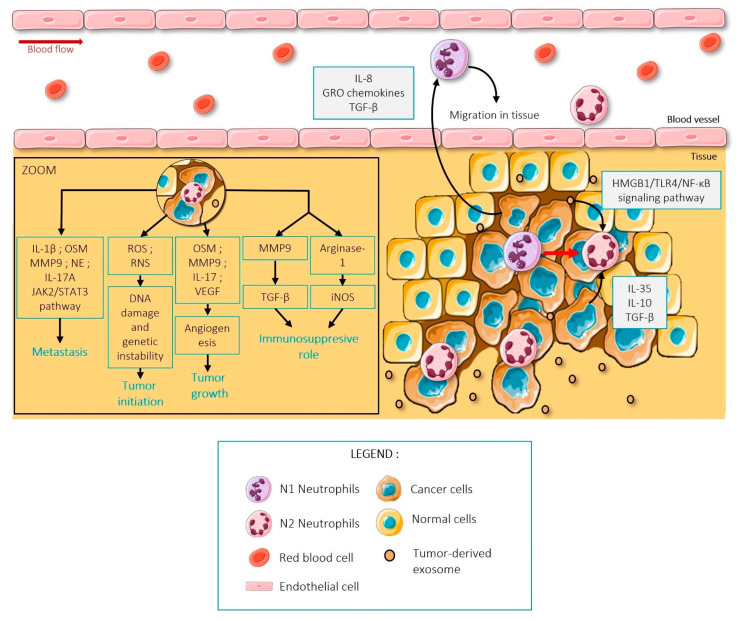
Summary of the role of N2 neutrophils during tumor development. Tumor cells can recruit circulating neutrophils and induce a switch from N1 to N2 neutrophils. Indeed, N2 neutrophils play an important role in several stages of cancer development, like tumor initiation and growth or metastasis formation by secreting several key molecules. Figure created using Servier Medical Art available at http://smart.servier.com/ (accessed on 15 September 2021).

In 2019, Wisdom and colleagues demonstrated that neutrophils could promote tumor resistance to radiation therapy in a genetically modified mouse model of sarcoma [69]. They do so by increasing a MAPK-regulated transcriptional program downstream of Kras and upregulating the expression of the AP-1 family transcription factors Fos and Jun, to promote cell proliferation [69]. Moreover, TANs are divided into two subpopulations: the N1, which have an anti-tumor behavior, and the N2, which have a pro-tumor one [70] (Figure 3). This pro-tumor profile of neutrophils can be favored by the cancer cells themselves [71,72]. It is described in the literature that tumor cells can differentially secrete cytokines such as IL-35, IL-10 or TGF-β to induce a switch from N1 to N2 neutrophil phenotype in the early stages of cancer [71,72]. Indirectly, tumor-derived exosomes can also polarize neutrophils into a N2 phenotype via HMGB1/TLR4/NF-κB signaling [73,74,75]. Therefore, tumor cells themselves can tip the N1-N2 balance to favor a pro-tumoral phenotype of neutrophils.

Pro-tumoral N2 neutrophils can produce reactive oxygen species (ROS) and reactive nitrogen species (RNS) to create DNA damage and genetic instability which can potentially initiate the tumor process [74,76]. They can also secrete mediators such as Oncostatin M (OSM), Matrix Metalloproteinase 9 (MMP9), IL-17 and VEGF into the tumoral microenvironment to induce angiogenesis and support tumor growth [72,77,78].

Pro-tumoral N2 neutrophils play a key role in the formation of metastases by secreting MMP9 and neutrophil elastase (NE) to favor the remodeling of the extracellular matrix necessary for tumor progression [77]. In addition, Li, S. et al. have shown that neutrophils can secrete IL-17A to activate the JAK2/STAT3 pathway to induce the epithelial-mesenchymal transition (EMT) [78]. Moreover, N2 neutrophils secrete inflammatory cytokines such as IL-1B and OSM to promote cancer cell migration and invasion [79]. Pro-tumoral N2 neutrophils are important allies that contribute to the tumor growth and spread through different mechanisms.

Interestingly, N2 neutrophils also have an immunosuppressive role [72,80,81]. They can eliminate tumor-infiltrating T lymphocytes (TIL) by activating TGF-β via MMP9 [80,81]. They also secrete high levels of arginase-1 and can activate the STAT3 and ERK pathways that lead to iNOS production and suppression of T-lymphocyte activation [70,72,81]. Arginase-1 and iNOS allow the transformation of L-arginine either into urea and L-Ornithine or Nitric Oxide (NO) and citrulline. Resulting in a decrease in L-Arginine levels, a molecule that is essential for the generation of CD3ζ75 [70,81,82]. CD3ζ is a chain of the CD3 complex that associates with the T cell receptor (TCR) and is essential for TCR signaling via the ITAM (immunoreceptor tyrosine-based activation motif) [82]. Without ITAM, the formation of a functional TCR and the proliferation of T lymphocytes are impacted [83]. In addition, extracellular vesicles derived from gastric cancer cells have been shown to induce PD-L1 expression on neutrophils to inactivate TLs [80,83].

Tumors are known to recruit macrophages and platelets to their microenvironment [14,84,85]. Indeed, high-infiltration of both macrophages and platelets can be correlated to a worsened prognosis [2]. Tumor-associated-neutrophils can influence macrophage polarization to tumor-associated-macrophages (TAM) by secreting Il-8, TNF-α and MPO [84,86,87,88]. These TAMs have T-cell immunosuppressive properties through PD-PD-L1 signaling and can upregulate Treg functions, thus contributing to the local immunosuppression that favors tumor growth [83,87,88].

Tumoral platelet infiltration is known to give tumors a survival advantage by degranulation, supporting both angiogenesis and tumor-growth [2]. It is logical to assume that neutrophils also interact with these cell types. Activated platelets express P-selectin, which can bind to neutrophils through the PSGL-1 receptor to create platelet-neutrophil aggregates that support metastasis by hiding cancer cells from shear forces in circulation [14,89,90]. Activated platelets can also secrete transforming growth factor beta (TGF-β1) to recruit more neutrophils to the tumoral site and increase T-cell immunosuppression [2,65,70,80].

## 5. NETs in Cancer Development

**Figure 4:**
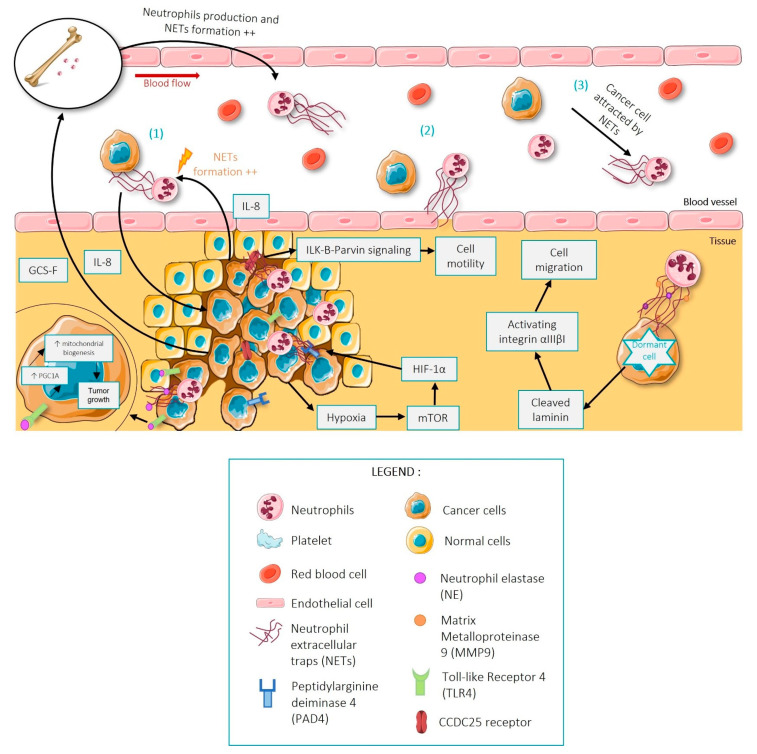
Schematic representation of the 3 main hypotheses of NETs in cancer (1) DNA network formed by NETs may trap circulating cancer cells at the site of dissemination. (2) DNA network formed by NETs may increase local vascular permeability that facilitates the extravasation of cancer cells into the surrounding tissues. (3) The extracellular DNA represented by NETs can act as a chemotactic factor to attract cancer cells. Figure created using Servier Medical Art available at http://smart.servier.com/ (accessed on 15 September 2021).

NETs can support a pro-tumoral role as they are believed to be involved in tumor growth, metastasis, and the awakening of dormant cancer cells [91] (Figure 4). This could explain why NETs are a marker of poor prognosis in cancer patients, especially in terminal cancer patients [92]. In 2016, Demers et colleagues demonstrated with PAD4-deficient mice that NETs are essential for promoting tumor growth [93]. However, the exact mechanisms involved remain unknown to date. In 2019, Yazdani et al. proposed that neutrophil elastase (NE) released by NETs activates TLR4 in cancer cells which results in upregulation of peroxisome proliferator-activated receptor gamma coactivator 1-alpha (PGC1A), a transcription coactivator that leads to increased mitochondrial biogenesis to provide additional energy for the tumor to accelerate its growth [94].

Interestingly, Zhang et al. found that the number of circulating NETs in peripheral blood correlated with disease stage in gastric cancer [95]. Indeed, Zhang et al. considered nucleosome-bound NE to indicate NET formation in serum and plasma of peripheral blood samples; these complexes were identified using capture ELISA [95].

In 2017, Park and colleagues demonstrated that blocking NET formation with DNase-I treatment reduced cancer invasion and prevented lung metastasis in mice [96]. Similarly, in colorectal cancer (CRC), it has been shown that increased NETs contribute to the development of CRC liver metastasis and that their digestion with DNase-I limits the increase in liver metastasis associated with NETs [97].

The exact mechanisms by which NETs are involved in metastasis remain somewhat unclear and controversial. Indeed, two initial hypotheses as to their involvement have been put forward: **(1)** that the DNA network formed by NETs may trap circulating cancer cells at the site of dissemination or **(2)** that they may increase local vascular permeability that facilitates the extravasation of cancer cells into the surrounding tissues [96]. Several studies support the first hypothesis that NETs facilitate the adhesion of circulating tumor cells to form metastases [98,99,100]. In 2020, Yang and colleagues demonstrated that NETs trap cancer cells but do not exert cytotoxicity on them [97]. NETs can increase the cancer cell’s proliferative and invasive capacity by triggering tumor IL-8 expression [97]. The over-expression of IL-8 can in turn activate neutrophils and generate NETs that promote metastasis [97]. 

A third hypothesis **(3)** regarding the role of NETs in metastasis formation has recently been put forward [101]. The extracellular DNA represented by NETs can act as a chemotactic factor to attract cancer cells, instead of simply entrapping them [101]. This has been shown in several mouse models and would occur via the CCDC25 receptor present on the surface of cancer cells [101]. In fact, the CCDC25 receptor then interacts with NETs to recruit ILK to initiate the β-parvin-RAC1-CDC42 cascade, which induces cytoskeletal rearrangement and tumor cell migration [101]. 

Last but not least, it has been shown in the literature that NETs are also involved in the awakening of so-called “dormant” or senescent cancer cells [102]. Tumor cells originating from the primary site can be disseminated in other tissues and remain dormant [102]. The stimuli that induce them to awaken are not well known. It has been described in mouse models that NE and MMP9 released by NETs can cleave laminin and induce the proliferation of dormant cancer cells by activating integrin α3β1 to mediate cell migration [102,103]. Thus, NETs seem to have many pro-tumor effects, but the exact molecular mechanisms remain elusive. 

However, the role of NETs in cancer progression remains controversial as anti-tumor effects have also been described [104]. For example, Arelaki and colleagues demonstrated in 2016 that NETs generated in vitro prevent the growth of colorectal cancer cells and primary myeloid leukemia cells, by inducing their apoptosis and/or inhibiting their proliferation [104]. These anti-tumor effects remain to date mostly unknown.

## 6. NETs and Cancer-Associated Thrombosis

Recently, researchers began to study the role of NETs in CAT, especially since CitH3 was shown to be present in the thrombi of cancer patients [105,106]. Numerous studies have corroborated the link between NETs and CAT, including a 2-year prospective study of 946 patients that shows that patients with elevated blood CitH3 levels had a higher cumulative incidence of VTE [107,108].

As we have previously stated, NETs can induce a pro-thrombotic and pro-coagulant state via multiple mechanisms. This pro-coagulant state can be amplified by the cancer cells themselves since they can secrete G-CSF (Granulocyte-Colony Stimulating Factor) in very large quantities [93,109,110]. G-CSF release induces a higher production of neutrophils and, as Demers and colleagues have shown, it also allows neutrophils to spontaneously create more NETs [109]. In addition, cancer cells themselves are also able to release PAD4 into their microenvironment, which promotes citrullination of histone 3 and chromatin de-condensation, leading to NET formation [95]. 

On the other hand, the tumor microenvironment can also increase the pro-thrombotic state. Indeed, tumors often grow faster than their blood supply, thus provoking hypoxia (which is characteristic of most solid tumors) [110]. McInturff et al. have shown that hypoxia favors the formation of NETs via the mammalian target rapamycin (mTOR) which regulates NET formation by post-transcriptional control of the expression of hypoxia-inducible factor 1 α (HIF-1α) [111]. 

Thus, NETs may be an attractive target for reducing CAT. Indeed, Boone and col-leagues have shown that the use of chloroquine reduces the hypercoagulability observed in pancreatic cancer by inhibiting NETs [112]. Furthermore, it has been shown in an orthotopic mouse model of breast cancer that the use of dunnione, a potent substrate of NAD(P)H quinone oxidoreductase 1, attenuates the pro-thrombotic state by inhibiting TF and NETs formation [113]. 

Elaskani et al. studied the NET-induced platelet aggregation and found that targeting the NET scaffold was not an effective strategy to reduce platelet activation [114]. More traditionally used anticoagulant and anti-platelet drugs like low molecular weight or un-fractioned heparin or direct-acting oral anticoagulants (Apixaban, dabigatran, rivaroxaban or endoxaban) continue to be the gold standard for thrombosis treatment in CAT [115,116]. Recently, the use of platelet P2RY12 inhibitors has been proposed to both prevent and treat TCIPA and CAT, but this application has not yet been validated in clinical trials [15,117].

## 7. Conclusions

We have endeavored to describe the current state of the literature on the relationship between neutrophils, thrombosis, and cancer. Neutrophilia is associated with worse prognosis in cancer patients and this increased neutrophil count has a direct impact on the development of cancer and CAT. Cancer cells themselves can participate in this relationship, creating a vicious circle that enhances both tumor growth and CAT. An important contributor to this relationship appears to be NET formation by neutrophils. NETS potentially sustain not only cancer growth, but also the development of cancer-associated thrombosis; yet the exact molecular mechanisms remain to be elucidated. We have shown that neutrophils play a key role in thrombus and CAT development; highlighting the necessity for further research to harness the power of neutrophils as new potential therapeutic or diagnostic targets in cancer.

## Abbreviations

ATP	Adenosine triphosphate
BPI	Bactericidal/permeability increasing protein
CAT	Cancer associated thrombosis
CCDC25	Coiled-Coil Domain Containing 25
CitH3	Citrullinated histone H3
CRC	Colorectal cancer
CXCL8/IL-8	Interleukin 8
EMT	Epithelial-mesenchymal transition
EPCAM	Epithelial cell adhesion molecule
ERK	Extracellular signal-regulated kinases
fMLP	N-formyl-Methionyl-leucyl-phenylalanine
G-CSF	Granulocyte-Colony Stimulating Factor
HIF-1α	Hypoxia-inducible factor 1 α
HMGB1	High-mobility group box 1
ICAM1/CD54	Intercellular Adhesion Molecule-1
ILK	Integrin-linked kinase
iNOS	Inducible nitric oxide synthase
ITAM	Immunoreceptor tyrosine-based activation motif
JAK2	Janus kinase 2
LFA1	Leukocyte-function-associated-antigen-1
LTB4	Leukotriene B4
MAC1	Macrophage-1 Antigen
MAPK	Mitogen-activated protein kinases
MMP9	Matrix Metalloproteinase 9
MPO	Myeloperoxidase
mTOR	Mammalian target rapamycin
TNFα	Tumor-necrosis-factor-α
VCAM1	Vascular Cell Adhesion Molecule-1
VEGF	Vascular Endothelial Growth Factor
MV	Microvesicles
NE	Neutrophil elastase
NET	Neutrophil extracellular traps
NF-κB	Nuclear factor-kappa B
nMV	Neutrophil derived MVs
NO	Nitric Oxide
OSM	Oncostatin M
P2RX1/P2X1	P2X purinoceptor 1
PAD4	Peptidylarginine deiminase 4
PDGF	Platelet derived growth factor
PD-L1	Programmed death-ligand 1
PECAM1	Platelet endothelial cell adhesion molecule-1
PGC1A	Peroxisome proliferator-activated receptor gamma coactivator 1-alpha
PMN	Polymorphonuclear cells
RNS	Reactive nitrogen species
ROS	Reactive oxygen species
STAT3	Signal transducer and activator of transcription 3
TAM	Tumor-associated macrophage
TAN	Tumor-associated neutrophils
TCIPA	Tumor cell induced platelet activation
TCR	T cell receptor
TF	Tissue Factor
TGF-β	Transforming growth factor beta
TIL	Tumor-infiltrating T lymphocytes
TLR4	Toll Like Receptor 4
TLs	T Lymphocytes
VEGF	Vascular endothelial growth factor
VTE	Venous thromboembolism
vWF	von Willebrand factor
MV	Microvesicles
NE	Neutrophil elastase
